# Morbidity and Mortality Patterns of Hospitalised Adult HIV/AIDS Patients in the Era of Highly Active Antiretroviral Therapy: A 4-year Retrospective Review from Zaria, Northern Nigeria

**DOI:** 10.1155/2012/940580

**Published:** 2012-09-17

**Authors:** Dimie Ogoina, Reginald O. Obiako, Haruna M. Muktar, Mukhtar Adeiza, Aliyu Babadoko, Abdulaziz Hassan, Isa Bansi, Henry Iheonye, Matthew Iyanda, Eric Tabi-Ajayi

**Affiliations:** ^1^Department of Medicine, Niger Delta University, PMB 071, Amassoma, Bayelsa State, Nigeria; ^2^Department of Medicine, Ahmadu Bello University Teaching Hospital, PMB 06, Zaria, Kaduna State, Nigeria; ^3^Department of Haematology, Ahmadu Bello University Teaching Hospital, PMB 06, Zaria, Kaduna State, Nigeria

## Abstract

*Background*. This study, undertaken in major tertiary hospital in northern Nigeria, examined the morbidity and mortality patterns of hospitalised adult HIV/AIDS patients in the HAART era. *Methods*. Between January 2006 and December 2009, admission records and causes of deaths of hospitalised medical HIV-infected patients were retrieved and analysed according to antiretroviral (ART) status. *Results*. Of the 207 HIV/AIDS patients reviewed, majority were newly diagnosed (73.4%), and most were hospitalised and died from various AIDS-defining illnesses, mainly disseminated tuberculosis and sepsis. Immune-inflammatory-reconstitution-syndrome, ART-toxicity and ART-failure, contributed to morbidity and mortality in patients receiving ART. Sixty six (31.9%) patients died, with higher mortality in males and in those with lower CD4-cell count, lower PCV, and shorter hospital stay. However, hospital stay ≤3 days and severe anaemia (PCV < 24%) were independent predictors of mortality. *Conclusion*. In the current HAART era, late presentation and tuberculosis continue to fuel the HIV/AIDS pandemic in Africa, with emerging challenges due to ART-related complications.

## 1. Background 

Nigeria is among the top ranked countries with high human immunodeficiency virus (HIV) burden worldwide, with about 3.1 million HIV-infected people and an estimated 215,000 HIV-related deaths in 2010 [1]. Many studies from Nigeria have described the critical role of HIV/acquired immunodeficiency syndrome (AIDS) as a cause of morbidity and mortality in both hospitalised and clinic-based adult patients [2–7].

These studies have revealed that most HIV-infected patients in Nigeria present late to health care facilities with features of advanced HIV-related disease such as weight loss and chronic diarrhoea [3–5], and pulmonary tuberculosis was found to be the most common cause of morbidity and mortality in majority of studies [2, 6, 7]. 

Currently, after more than eight years of availability of highly active antiretroviral therapy (HAART) in Nigeria, there is still a dearth of studies determining the morbidity and mortality patterns of hospitalised adult HIV/AIDS patients in relation to antiretroviral therapy (ART) status. Although few studies have evaluated the clinical and laboratory presentations of ART-related drug toxicities among out-patients [8, 9], data is currently lacking on the spectrum of ART-related complications such as immune reconstitution inflammatory syndrome (IRIS) and antiretroviral (ARV) drug toxicities in hospitalised patients from Nigeria. Furthermore, there is paucity of data on clinical and laboratory predictors of mortality in hospitalised adult HIV/AIDS patients from Nigeria. In this study, undertaken in a major tertiary hospital in northern Nigeria, we sought to describe the morbidity and mortality patterns of hospitalised adult HIV/AIDS patients in relation to their ART status. The clinical and demographic variables associated with mortality were also ascertained. The results obtained may be useful to health care providers, policy makers, and other stakeholders in Nigeria in the provision of required diagnostic and therapeutic facilities in hospitals according to prevalent diseases, and in the preemptive management HIV-related clinical complications, as well as in the prevention of HIV-related morbidities and mortalities.

## 2. Methods

### 2.1. Design

A retrospective cohort analysis of routinely collected medical records of adult (>13 yrs) nonpregnant HIV/AIDS patients admitted into medical wards of Ahmadu Bello University Teaching Hospital (ABUTH) between January 2006 and December 2009 was undertaken using a standardized data extraction form. Only hospitalised patients with complete clinical details indicating diagnosis and clinical outcome were included. The demographic, clinical, and laboratory parameters of patients at the time of last hospitalisation were retrieved and analysed. Clinical diagnoses of prior hospitalisations during study period were documented when available. Based on ART status at last hospitalisation, patients were categorised into two groups, including group 1 (no previous ART experience) and group 2 (previous ART experience). Group 1 was defined as patients who had no prior history of receiving ART (based on patients account and available records) at the time of hospitalisation, while group 2 was defined as patients who had a prior history of receiving ART for whatever duration. Adherence to ART and use of Cotrimoxazole prophylaxis were not assessed as this information was not available in the admission medical records for most of the patients. 

### 2.2. Setting

The study was undertaken in ABUTH, Zaria, a 700-bed referral hospital in Zaria, Kaduna state, Northern Nigeria. The HIV/AIDS treatment centre, situated within the hospital premises, offers comprehensive HIV treatment and care, including diagnosis of opportunistic infections, to adults, pregnant women, and children. Over 5000 HIV-infected nonpregnant adults are currently under care in the outpatient clinic, and sick patients are often admitted into the medical wards, managed by teams led by consultant physicians of various specialities. The HIV/AIDS treatment centre is supported by the US Presidents Emergency Plan for AIDS Relief (PEPFAR). 

### 2.3. Diagnosis

The diagnoses of HIV/AIDS-related events, ARV drug toxicities, and ART failure were based on WHO 2006 guidelines [10]. However, viral loads and serial CD4 counts were not available for majority of patients, so the diagnosis of ART failure was mainly clinical. ARV drug toxicities was defined as clinically symptomatic ARV drug-related adverse drug reactions presenting after commencement of HAART, with exclusion of other causes of presentation. 

A case of IRIS was defined as a patient with unexpected deterioration in clinical condition with signs and symptoms of inflammation/infection soon after commencing ART (<6 months of regular ART). IRIS cases were classified as paradoxical or unmasking according to established clinical guidelines [11–13]. 

Aetiological diagnosis was based on compatible clinical presentation, response to therapy, or confirmatory investigations, all in accordance WHO 2006 guidelines for defining HIV/AIDS-related events [10]. Briefly, the diagnosis of pulmonary tuberculosis (TB) required compatible clinical features and suggestive chest X-ray (CXR) findings or positive sputum acid fast bacilli. Extra-pulmonary TB was defined by compatible clinical features and suggestive investigations such as spinal X-rays (for spinal TB) and typical findings of pleocytosis with mononuclear predominance, elevated protein, and low glucose on cerebrospinal/pericardial/pleural fluid analysis or a positive AFB staining (for TB meningitis, pericarditis, and pleuritis). Tissue histopathology confirmed diagnoses of TB adenitis, Kaposi's sarcoma, HIV-nephropathy, non-Hodgkin's lymphoma, and glioblastoma multiforme. Cerebral toxoplasmosis was defined by compatible clinical features and positive IgG toxoplasma serology or suggestive brain imaging findings or response to antitoxoplasma therapy. Primary CNS lymphoma was diagnosed by compatible history, negative toxoplasma serology, and ring enhancing lesion on MRI with cerebral oedema. Cryptococcal meningitis was defined by compatible history and positive CSF Indian ink stain/Cryptococcal antigen or response to fluconazole therapy. Cryptosporidiosis was confirmed by stool modified Ziehl Nielsen's stain. Patients presenting with focal deficits that could be investigated or treated to establish aetiological diagnosis were classified as stroke-like states of unknown aetiology. Sepsis definitions were based on the ACCP/SCCM Consensus Conference [14]; sepsis was present if there was a presumed or confirmed infection, associated with at least two of the following: tachycardia >90 beats/min; tachypnea >30 cycles/min; fever (>38°C) or hypothermia (<36°C); leukocytosis (>12,000/mm^3^) or leukopenia (<4,000/mm^3^) or the presence of more than 10% immature forms. Because majority of blood cultures were negative, diagnosis of sepsis and typhoid fever was mainly clinical. 

The treatment of all opportunistic infections and use of HAART was according to the Nigerian National HIV adult treatment guidelines [15] as well as the WHO 2006 guidelines [10]. All laboratory investigations were done on hospitalisation or just before hospitalisation. All CD4 cell counts included in study analyses were either done on hospitalisation (mainly for group 1 patients) or within the previous 3 months before hospitalisation (mainly for group 2 patients). According to national guidelines, CD4 cell counts are to be repeated 3–6 monthly [15].

### 2.4. Definition of Outcomes

Survival outcomes (i.e., died or survived) were based on last contact with physician during hospitalisation. All discharged cases, cases of “discharged against medical advice” (DAMA), and transferred cases were classified as survived. Causes of death, filled into standardized death certification forms by attending physician at time of death, were discussed, reviewed, and validated at weekly mortality meetings. Unfortunately, permission for postmortem was not given for most patients in view of incongruous cultural/religious beliefs. 

The ABUTH Institutional Review Board gave approval for the study. 

### 2.5. Statistical Analysis

Statistical analysis was undertaken using SPSS 17. Descriptive statistics were represented as median and interquartile range (IQR). Differences in variables by ART status were sought by Mann Whitney test and chi-square test as appropriate. Demographic and available laboratory data were compared according to survival status (i.e., died or survived). An unconditional binary logistic regression analysis checked for model fitness and interactions, and represented in odds ratio (OR) with 95% confidence interval (CI) was used to determine independent predictors of mortality. For regression analysis, we categorised age into young adults (15–45 yrs) and middle age/elderly (>45 yrs), PCV into severe anaemia (PCV < 24%,) and mild or no anaemia (PCV ≥ 24%), CD4 cell count into very severe immunosuppression (CD4 ≤ 50 cells/ul) and low or normal (CD4 > 50 cells/ul), symptom duration into acute (<30 days) and chronic (≥30 days), and hospital stay into ≤3 days and >3 days. Gender (male/female) and ART status (group 1/group 2) were also included in the regression analysis. Differences in survival between group 1 and 2 patients were represented in a Kaplan-Meir survival curve. *P* < 0.05 was considered statistical significant for all analyses. 

## 3. Results 

### 3.1. Characteristics of Studied Population

A total of 207 HIV/AIDS patients, representing 5.9% of 3464 adult medical admissions, and consisting of 152 (73.4%) group 1 and 55 (26.6%) group 2 patients, were studied. The demographic and clinical characteristics of patients according to ART status are shown in Table 1.

The group 2 patients were older than the group 1 patients (40 versus 36 yrs, *P* = 0.012), but other demographic and clinical variables did not significantly differ by ART status (Table 1). In patients receiving ART (group 2), duration of HAART ranged from 10 days to 8 years (median 8 months), and majority (94.5%) were on first line drugs, including Zidovudine/Lamivudine/Nevirapine (*n* = 22), Stavudine/Lamivudine/Nevirapine (*n* = 18), Zidovudine/Lamivudine/Efavirenz (*n* = 5), and Tenofovir/Emtricitabine/Nevirapine or Efavirenz (*n* = 7). Only three patients were on second-line ART including Zidovudine/Tenofovir/Emtricitabie/Liponavir-Ritonavir. 

In both ARV groups, majority were heterosexuals (92.8%), males (52.9%), and ever married (56.5%), and most were admitted with anaemia-PCV < 30% (60%) and severe immunosuppression CD4 ≤ 200 cells/ul (76.2%).

### 3.2. Gender Differences in Baseline Characteristics

With regard to gender, median ages (IQR) of males were significantly higher than those for females in both group 1 (38 years (32, 46) versus 32 years (27, 38), *P *< 0.0001, *Z *= −3.58) and group 2 (42 years (38, 51) versus 35 years (30, 42), *P *= 0.009, *Z *= −2.59) patients. All other clinical and laboratory variables were comparable by gender (*P* > 0.05) in both ART groups (data not shown).

### 3.3. Diagnoses of Patients

The clinical diagnoses of patients according to ART status are shown in Table 2. Of the 152 group 1 patients, 148 (97.4%) were admitted on account of HIV-related diagnosis, while 4 (2.6%) were HIV-unrelated. Of the 55 group 2 patients, 24 (43.6%) had ART failure, 15 (27.3%) had IRIS (9 with paradoxical reactions and 6 unmasking), 8 (14.5%) had ART-related toxicities, 4 (7.3%), had HIV-unrelated diagnoses, and in another 4 (7.3%), the diagnoses were unclassified. All four cases of AZT-induced anaemia had PCV < 15% (grade 4-toxicity) [7]. All cases of IRIS and ART-related toxicities were receiving first line drugs. 

Although a variety of clinical manifestations were observed, tuberculosis, sepsis, and chronic diarrhoea were the most common diagnoses in both ART groups. The various types of TB and other specific aetiological diagnoses are summarised in Table 3. The common causes of morbidity in the 24 ART failure cases included TB (*n* = 7), sepsis (*n* = 4), chronic diarrhoea (*n* = 4), and non-TB pneumonia (*n* = 3).

The 9 paradoxical IRIS reactions included DTB (*n* = 6), PTB (*n* = 2) and disseminated Kaposi's sarcoma (*n* = 1), and the 6 unmasking IRIS reactions included DTB (*n* = 3), and one each of cerebral toxoplasmosis, cryptococcal meningitis, and multidermatomal herpes zoster.

### 3.4. Gender Differences in Clinical Diagnoses

The male to female ratio in diagnoses is shown in Table 2. In diagnoses where males and females were represented, more males were admitted for KS, cerebral toxoplasmosis, stroke-like states, crytpococcal meningitis, and AIDS encephalopathy, whereas more females were admitted for TB, Non-TB pneumonia, viral meningoencephalitis, and non-Hodgkin's lymphoma. Seven (87.5%) of the eight ARV drug toxicities were males. However, these observed gender differences were not statistically significant (*P* > 0.05 for all analyses by chi-square). 

### 3.5. Diagnoses at Prior Hospitalisations

There were 32 documented prior hospitalisations during study period, out of which 19 (59.3%) were in group 1 patients and 13 (30.7%) were in group 2 patients. Group 1 patients had prior hospitalisations for pulmonary tuberculosis (*n* = 3), chronic diarrhoea (*n* = 4), typhoid fever (*n* = 2), lobar pneumonia (*n* = 1), and chicken pox (*n* = 1), as well as for severe hypertension (*n* = 1) and for surgical-related diseases (*n* = 7) such as appendectomy, ruptured ectopic pregnancy, haemorrhoids, and accidental injuries. The prior hospitalisations diagnoses in group 2 patients included pulmonary tuberculosis (*n* = 5), diarrhoea (*n* = 2), typhoid fever (*n* = 1), hypertensive heart failure (*n* = 1), and Zidovudine-induced anaemia (*n* = 2), as well as for acute severe asthma (*n* = 1) and obstructive urinary symptoms due to benign prostatic hypertrophy (*n* = 1). Other than re-admissions for chronic diarrhoea in some patients, no other diagnoses during prior hospitalisations were similar to the diagnoses during the last hospitalisation.

### 3.6. Outcome and Causes of Death

Of the 207 admitted patients, 112 (54.1%) were discharged, 27 (13%) were “DAMA.” 2 (1%) were transferred and 66 (31.9%) died. The 66 cases of mortality consisted of 47 (30.9%) of 152 group 1 and 19 (34.5%) of 55 group 2 patients. 

The various causes of death are listed in Table 4. Again, tuberculosis was the most common cause of death in both groups. Of the 19 deaths in group 2, 9 (47.4%) were cases of ART failure, 8 (42.1%) were IRIS cases, and one each were cases of ARV drug toxicity (NVP hepatic failure) and non-HIV related end-stage renal failure.

### 3.7. Variables Associated with Mortality

Differences in clinical variables in relation to outcome (died or survived) are summarised in Table 5. Patients that died had longer symptom duration, shorter hospital stay, lower PCV, and lower platelet counts compared with patients that died. Males were also more likely to have died than females. 

The predictors of mortality are summarised in a univariate and a multivariate analysis as shown in Table 6. On univariate analysis, male sex, CD4 < 50 cells/ul, PCV < 24%, and hospital stay ≤3 days were significantly associated with mortality. However, following a multivariate regression analysis, short hospital stay (≤3 days) and severe anaemia (PCV < 24%) were the only variables independently associated with mortality. 

**Figure 1:**
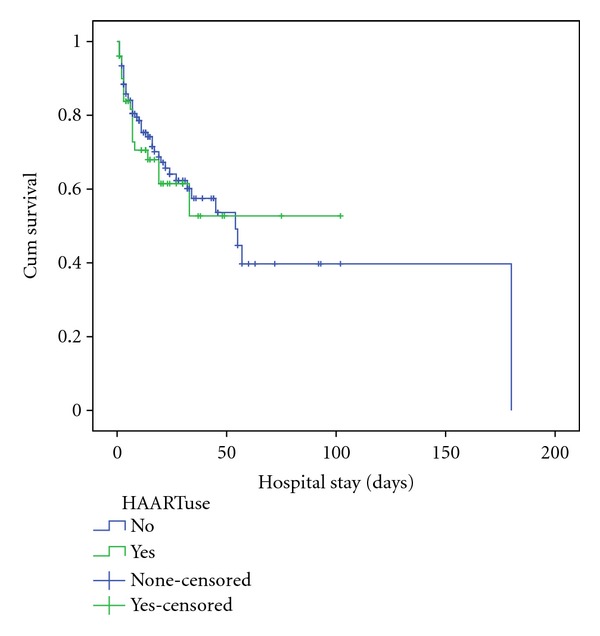
Survival after hospitalisation in relation to antiretroviral status—no significant difference in survival rates were observed between both ART groups (Log rank *P *= 0.65, Breslow *P *= 0.44). Survival rates dropped sharply in both ART groups within the first 10 days of hospitalisation.

The Kaplan-Meir survival curves of both ART groups shown in Figure 1 revealed a sharp drop in survival rates in both groups within the first 10 days of hospitalisation. There were no significant differences in survival rates on comparisons of both ART groups (Log rank *P* = 0.65, Breslow *P* = 0.44).

## 4. Discussion 

The results of this study has shown that in the current era of HAART in Nigeria, majority of the hospitalised HIV-infected patients are heterosexuals of young productive age and that most of the newly diagnosed HIV-infected patients as well as patients receiving ART are hospitalised on account of AIDS-defining illnesses characterised by severe immunosuppression and anaemia. These findings possibly reflect late HIV diagnosis and delay in initiation of HAART. It is worrisome that even with the provision of free ARV drugs in many parts of Sub-Saharan African many HIV/AIDS patients from this region still suffer from advanced HIV-related diseases [16], whereas in the developed world, morbidity is mainly due to HIV-unrelated diseases [17, 18]. To facilitate early HIV diagnosis and early initiation of HAART, all stakeholders in the region must make concerted effort to expand and implement voluntary counselling and testing as well as the provider-initiated testing and counselling, even as new strategies are developed to detect early infection for prompt initiation of HAART, when necessary. 

Although the morbidity and mortality of HIV/AIDS in Nigeria are known to predominantly affect females [1], our study revealed that more males than females were admitted and died of HIV/AIDS. Higher hospitalisation of males than females has also been reported in other hospital-based studies of HIV-infected and noninfected populations from northern Nigeria [2, 19, 20]. In our environment, the family economic power rest with the men and it is likely than women is underrepresented because very sick women either never get to the hospital or die before decisions are made to take them to the hospital. We cannot tell if the observed male preponderance in some aetiological diagnoses was also due to these reasons. It is also not clear why our newly diagnosed HIV-infected patients without ART experience were younger that the patients receiving ART, but it may probably be due to the time lag between HIV diagnosis and initiation of ART. However, in agreement with other studies [2, 3, 6, 21], our male patients were older than the females irrespective of ART status. This finding has been attributed to earlier sexual maturity in females and, therefore, earlier risk of acquisition of HIV infection in females than males [22].

In studied participants, we observed a variety of HIV-related and unrelated manifestations, but tuberculosis, followed by sepsis and chronic diarrhoea, were the most common causes of morbidity in both groups of patients. This spectrum of clinical presentations is similar to that reported in an earlier study of hospitalised HIV/AIDS patients in our region in the pre-HAART era [23], as well as studies from other parts of Nigeria [2, 3, 6, 7, 24] and other resource-limited settings [25, 26] in the HAART era. In view of the heightened risk of TB coinfection in HIV-infected patients, there is a critical need to foster and strengthen TB/HIV collaborative services in Nigeria, like in many other Sub-Saharan countries where both infections are endemic. Consistent with other studies [27, 28], Kaposi's sarcoma (KS) was the most common malignancy and cerebral toxoplasmosis was the most common cerebral mass lesion in our patients, although aetiological causes for some cases of focal deficits could not be established. Toxoplasmosis and HHV8 infection, the causative agent for KS, are both endemic in Nigeria [29, 30], and in our region, HHV8 infection have been shown to be more prevalent in HIV-infected patients than HIV negative individuals [31]. Consequently, government and other stakeholders in Nigeria ought to develop locally suitable and comprehensive preventive and management guidelines for these endemic infections, especially in HIV-infected patients. 

Possibly reflecting the benefits of HAART in reducing rates of hospitalisation [32], only few of our hospitalised patients were receiving ART. The majority of these patients were admitted for AIDS-defining illnesses due to ART treatment failure as well as for direct ART-related complications such as IRIS and drug toxicities. AZT-induced severe anaemia requiring blood transfusions was the most common reason for admission. Hence, patient on this drug ought to be closely monitored for anaemia as well as for other related ART adverse reactions such as lactic acidosis, peripheral neuropathy, and lipodystrophy which have been reported among outpatients in our region [9]. As efforts are made to scale up HAART to reach more than 500,000 individuals with advanced HIV in Nigeria who are currently not receiving HAART [1], stakeholders in Nigeria must begin to recognise and tackle challenges such as ARV drug resistance and poor ART adherence known to fuel ART treatment failure, even as future studies investigate the risk factors for IRIS and ARV drug toxicities in adult Nigerians. 

Although some differences in study design exist, the overall mortality rate of 31.8% observed in our study population is comparable to the mortality rates of 26 to 40% reported in other studies of hospitalised HIV/AIDS patients during the HAART era from Nigeria [2, 7, 24, 33] and other resource-limited settings [21, 25, 26, 34]. Like in our study, late presentation and advanced HIV as reflected in low CD4 cell counts were factors implicated for the reported high mortality rates in these studies. Although not reaching statistical significance, possibly due to small sample size, the median duration of HAART before death in our ART-experienced patients was 3 months as compared to 10 months for those that survived. This finding is in agreement with other studies that have shown that mortality is highest in the first 3 months of HAART [35, 36]. 

In our patients, PCV was found to be independently associated with mortality with a six-fold risk of mortality in patients with severe anaemia. This finding corroborates studies from other developing countries [35, 37], as well as developed countries where anaemia was also found to be an independent predictor of mortality in HIV/AIDS patients [38]. Anaemia is marker of progressive HIV disease as it is a prominent feature of most opportunistic infections complicating HIV-disease, including TB [39]. In the setting of HIV/AIDS, anaemia may result from micronutrient deficiencies, immunological myelosuppression, impaired erythropoietin production, and blood loss from intestinal opportunistic disease, among other causes [39]. Since assessment of PCV is simple and rapid, routine screening for anaemia in resource poor settings can be a cost-effective tool for identifying high-risk HIV/AIDS patients for closer followup and targeted interventions. 

There are limitations to our study. First, since the study was retrospectively designed, detailed clinical and laboratory variables were not available for all patients. However, we believe the missing data did not significantly affect the major outcomes of the study since our findings were comparable to those within and outside Nigeria. Data quality can be improved by future prospective studies from Nigeria to clarify the independent associations, if any, between variables such as CD4 and platelet counts, and mortality of hospitalised HIV/AIDS patients in Nigeria, as shown by studies from other parts of Africa [35]. 

Second, we could not confirm the causes of deaths in most patients because of lack of permissions for autopsies. However, the listed causes of death were standardized and further validated during mortality reviews. The aetiological causes of sepsis could also not be confirmed by positive blood cultures possibly because most patients often practise self-medication or receive antibiotics elsewhere before hospitalisation, making cultures negative. However, culture of microorganisms such as mycobacteria and other atypical microorganisms reported to cause sepsis in HIV-infected patients [40] are not routinely done in our centre due to resource constraints. Third, since serial viral loads were not available for most patients on HAART, it is plausible that some cases of virological failure without concomitant clinical failure would have been missed. Furthermore, in the absence of reliable records, it was impossible to decide from patient's retrospective records if poor ART adherence contributed in any way to treatment failure. Lastly, given that outcome measure for our study depended on the survival status as at last contact with our patient in the hospital, we cannot exclude an underrepresentation of mortality rate as some DAMA patients might have died outside our hospital. 

## 5. Conclusions 

This study undertaken in a major tertiary hospital in Northern Nigeria during the HAART era has shown that majority of hospitalised HIV/AIDS patients are heterosexuals of young productive age, males and newly diagnosed HIV-infected patients with no previous ART experience. Most patients were admitted on account of AIDS defining illness such as disseminated TB and sepsis, with features of severe immunosuppression and anaemia. The study data also revealed that mortality was high due to late presentation and advanced disease, and that there was a six-fold risk of mortality in those with severe anaemia. To combat the high morbidity and mortality associated with HIV/AIDS in developing countries such as Nigeria, strategies for early HIV diagnosis, prompt initiation of HAART, prevention of TB co-infection in HIV, and early recognition of danger signs such as low PCV must be initiated, implemented, and strengthened as necessary. 

## Figures and Tables

**Table 1:** Baseline characteristics of hospitalised HIV/AIDS patients in relation to antiretroviral therapy status.

Characteristics	No HAART *N* = 152	Receiving HAART *N* = 55	All patients *N* = 207
Age in years-median (IQR)	35 (29, 43)	40 (34, 48)	36 (30, 45)
Minimum–Maximum	15–68	23–60	15–68
Gender *N* (%)			
Male	77 (50.7)	32 (58.2)	109 (52.7)
Female	75 (49.3)	23 (41.8)	98 (47.3)
Marital status *N* (%)			
Ever married	120 (78.9)	44 (80.4)	164 (79.3)
Never married	32 (21.1)	11 (19.6)	43 (20.7)
Occupation *N* (%)			
Professionals/Civil servants	37 (24.3)	14 (25.5)	51 (24.6)
Unemployed housewives	52 (34.2)	12 (21.8)	64 (30.9)
Artisans	20 (13.2)	7 (12.7)	27 (13)
Students	13 (8.6)	6 (10.9)	19 (9.3)
Traders/Business	11 (7.2)	8 (14.5)	19 (9.3)
Farmer	6 (3.9)	2 (3.6)	8 (3.9)
Others	13 (8.6)	7 (12.7)	20 (9.7)
Risk factors for HIV *N* (%)			
Heterosexual	142 (93.4)	50 (90.9)	192 (92.8)
Homosexual	3 (2.0)	2 (36.4)	5 (2.4)
Multiple sexual partners	21 (13.8)	13 (23.6)	34 (16.4)
Blood transfusion	7 (4.6)	3 (5.5)	10 (4.8)
Symptom duration in days			
Median (IQR)	21 (14, 90)	60 (14, 90)	30 (14, 90)
Hospital stay in days			
Median (IQR)	14 (5, 24)	14 (7, 30)	14 (7, 28)
Packed cell volume			
Median (IQR)	27 (20, 33)	24 (17, 34)	27 (19, 34)
PCV levels *N* (%)			
<24%	22 (31.9)	19 (48.7)	41 (38%)
24–29%	20 (29.0)	4 (10.3)	24 (22.2%)
≥30%	27 (39.1)	16 (41.0)	43 (39.8%)
Total WBC count			
Median (IQR)	5.0 (3.7, 6.5)	4.7 (3.9, 6.1)	4.9 (3.7, 6.5)
Platelet count-Median (IQR)	246 (145, 331)	230 (157, 271)	233 (142, 310)
CD4 cell count-Median (IQR)	136 (5, 201)	137 (56, 190)	136 (56, 199)
CD4 levels *N* (%)			
<50	11 (23.9)	9 (23.7)	20 (23.8%)
50–200	23 (50.0)	21 (55.2)	44 (52.4%)
201–350	7 (15.2)	6 (15.8)	12 (14.3%)
≥351	5 (10.9)	2 (5.3)	7 (8.3%)

NB—*N*: number; IQR: interquartile range; WBC: white blood cell; PCV: packed cell volume.

No statistical significant differences were observed in demographic and laboratory variables between HAART experienced and ART naive patients (*P* > 0.05, all analyses).

**Table 2:** Clinical diagnoses of hospitalised HIV/AIDS patients in relation to antiretroviral therapy status and gender.

Diagnosis on presentation	ART status (*N*%)		Total (*N*%)	M/F
	No ART	Receiving ART		
Tuberculosis	53 (34.9)	16 (29.1)	69 (33.3)	0.87/1
Sepsis	15 (9.2)	6 (7.3)	21 (10.1)	1.1/1
Chronic diarrhoea	8 (5.3)	6 (7.3)	14 (6.8)	1.8/1
Typhoid fever	8 (5.3)	—	8 (3.9)	1.7/1
Non-TB Pneumonia	8 (5.3)	3 (5.5)	11 (5.3)	0.4/1
Disseminated Kaposi's sarcoma	7 (4.6)	1 (1.8)	8 (3.9)	2/1
Cerebral toxoplasmosis	6 (3.9)	1 (1.8)	7 (3.4)	2.3/1
Viral meningoencephalitis	5 (3.2)	1 (1.8)	6 (2.9)	0.7/1
Demyelinating polyneuropathy	5 (3.2)	—	5 (2.4)	0.7/1
Cryptococcal meningitis	4 (2.6)	1 (1.8)	5 (2.4)	4/1
AIDS encephalopathy	3 (2%)	—	3 (1.4)	2/1
Non-Hodgkin's lymphoma	3 (2%)	—	3 (1.4)	0.5/1
Acute gastroenteritis (Food poisoning)	2 (1.3%)	2 (3.6)	4 (1.9)	0.7/1
Steven Johnson's syndrome	2 (1.3)	2 (3.6)	4 (1.9)	1/1
Herpes zoster	2 (1.3)	1 (1.8)	3 (1.4)	0.5/1
Acute bacterial meningitis	2 (1.3)	—	2 (0.9)	2/0
Wasting syndrome	2 (1.3)	—	2 (0.9)	1/1
Acute viral hepatitis (HBsAg positive)	2 (1.3)	—	2 (0.9)	1/1
HIV nephropathy	2 (1.3)	—	2 (0.9)	1/1
Candidiasis (esophageal; disseminated)	2 (1.3)	—	2 (0.9)	1/1
Vacuolar myelopathy	1 (0.7)	—	1 (0.5)	0/1
Disseminated herpes simplex	1 (0.7)	—	1 (0.5)	0/1
Severe malaria	1 (0.7)		1 (0.5)	0/1
Dilated cardiomyopathy	—	1 (1.8)	1 (0.5)	0/1
Primary CNS lymphoma	1 (0.7)	—	1 (0.5)	1/0
Glioblastoma multiforme	1 (0.7)	—	1 (0.5)	1/0
Stroke-like state? cause	3 (2)	3 (5.5)	6 (2.9)	3/1
Primary liver cell carcinoma	1 (0.7)		1 (0.5)	1/0
Zidovudine-related severe anaemia	—	4 (7.3)	4 (1.9)	2/1
Nevirapine-induced hepatoxicity	—	2 (3.6)	2 (0.9)	2/0
Nevirapine-induced Steven's Johnson syndrome		2 (3.6)	2 (0.9)	2/0
Hypertensive heart failure	—	2 (3.6)	2 (0.9)	1/1
Hypertensive renal failure	1 (0.7)	2 (3.6)	3 (1.4)	0.5/1
Hypertensive haemorrhagic stroke	1 (0.7)		1 (0.5)	0/1
Peripartum cardiac failure	1 (0.7)		1 (0.5)	0/1

NB—M/F is the ratio of number of male patients divided by female patients for each diagnosis in the total population.

**Table 3:** Types of tuberculosis and specific aetiological diagnoses in patients in relation to antiretroviral therapy status.

Diagnoses and specific aetiologies	ART status *N*%		Total
	No HAART	Receiving-HAART
Types of tuberculosis			
Disseminated (mainly nodes and lungs)	30	12	42
Pulmonary	10	2	12
Spinal	6	—	6
Meningitis	5	—	5
Pericardial effusion	—	1	1
Pleural effusion	1	1	2
Peritonitis	1	—	1
Causes of diarrhoea			
* Salmonellosis *	1	—	1
* Giardia Lamdlia *	2	—	2
* Strongyloides stercoralis *	1	—	1
* Cryptosporidium parvum *	1	—	1
Unknown*	3	6	9
Causes of pneumonia			
* Streptococcus pneumonia *	2	1	3
* Klebsiella pneumonia *	1	—	1
Pneumocystis jiroveci**	—	1	1
Unknown*	5	1	6

NB—*unknown implied that stool/sputum culture was negative or not done.

**Diagnosis of *pneumocystis jiroveci* was clinical.

**Table 4:** Causes of death in hospitalised HIV/AIDS patients in relation to antiretroviral therapy status.

Causes of death	ART status (*N*%)		Total
	No HAART	Receiving-HAART
(1) DTB	14 (29.8)	6 (31.6)	20 (29.9)
(2) PTB with respiratory failure	3 (6.4)	1 (5.3)	4 (6.0)
(3) TB meningitis	2 (4.3)	—	2 (3.0)
(4) Bone marrow TB	1 (2.1)	—	1 (1.5)
(5) Sepsis	9 (19.1)	4 (21.1)	13 (19.7)
(6) Viral meningoencephalitis	2 (4.3)	1 (5.3)	3 (4.5)
(7) Cryptococcal meningitis	2 (4.3)	1 (5.3)	3 (4.5)
(8) Disseminated Kaposi's sarcoma	2 (4.3)	1 (5.3)	3 (4.5)
(9) Non-Hodgkin's lymphoma	2 (4.3)	—	2 (3.0)
(10) Hypovolaemic shock from gastroenteritis	2 (4.3)	—	2 (3.0)
(11) Stroke-like state? cause	2 (4.3)	1 (5.3)	3 (4.5)
(12) Acute bacterial meningitis	1 (2.1)	1 (5.3)	2 (3.0)
(13) Severe pneumonia	1 (2.1)	1 (5.3)	2 (3.0)
(14) Hepatic failure	1 (2.1)	1 (5.3)	2 (3.0)
(15) Disseminated candidiasis	1 (2.1)	—	1 (1.5)
(16) PLCC	1 (2.1)	—	1 (1.5)
(17) Pulmonary embolism-Vacuolar myelopathy	1 (2.1)	—	1 (1.5)
(18) End stage renal failure	1 (2.1)	1 (5.3)	2 (3.0)

**Table 5:** Comparisons of demographic and clinical variables in relation to survival outcome of HIV/AIDS patients.

Variables	Survival status		*P* value
	Died	Survived
Age in years			
Median (IQR)	38 (30, 46)	35 (30, 45)	0.29 (NS)
Gender (*N* %)			
Male	42 (38.5%)	67 (61.5%)	0.03
Female	24 (24.5%)	74 (75.5%)	
Symptom duration in days—			
Median (IQR)	60 (15, 120)	30 (14, 90)	0.018
Hospital stay in days			
Median (IQR)	7 (3, 18)	17 (10, 31)	<0.0001
PCV			
Median (IQR)	22 (16, 29)	28 (20, 35)	0.012
Platelets count			
Median (IQR)	129 (85, 252)	242 (186, 312)	0.016
CD4 cell count			
Median (IQR)	45 (21, 237)	140 (60, 194)	0.18 (NS)
ART duration (yrs)			
Median (IQR)	0.25 (0.17, 3.0)	0.8 (0.28, 2.0)	0.67 (NS)

NB—NS: not significant; PCV: packed cell volume; IQR: interquartile range.

**Table 6:** Variables associated with mortality in hospitalised HIV/AID patients.

Variable*	Univariate		Multivariate	
	OR (95% CI)	*P* value	AOR (95% CI)	*P* value
15–45 yrs	0.84 (0.54–1.33)	0.47	0.85 (0.12–6.22)	0.87
Male	1.57 (1.03–2.39)	0.03	5.3 (0.96–28.9)	0.06
Receiving ART	1.12 (0.72–1.73)	0.62	4.8 (0.8–28.4)	0.09
Symptom duration ≥ 1 month	1.42 (0.90–2.23)	0.13	2.2 (0.42–11.8)	0.35
Hospital stay ≤ 3 days	2.71 (1.94–3.79)	<0.0001	13.8 (1.1–178)	0.045
CD4 count < 50	3.2 (1.28–8.0)	0.012	2.6 (0.5–13.8)	0.27
PCV < 24%	2.1 (1.15–3.87)	0.015	5.7 (1.2–26.7)	0.029

NB—*Variable lists are all reference values. OR: odds ratios; AOR: adjusted odds ratio; CI: confidence interval. [Model goodness of fit- *P* = 0.68].
